# Detection and Quantification of Some Ethanol-Producing Bacterial Strains in the Gut of Mouse Model of Non-Alcoholic Fatty Liver Disease: Role of Metformin

**DOI:** 10.3390/ph16050658

**Published:** 2023-04-27

**Authors:** Mohamed Abouelkheir, Ibrahim Taher, Amira S. R. Eladl, Dalia A. Shabaan, Mona F. M. Soliman, Ahmed E. Taha

**Affiliations:** 1 Department of Pharmacology and Therapeutics, College of Medicine, Jouf University, Sakaka 72388, Saudi Arabia; 2 Department of Pharmacology, College of Medicine, Mansoura University, Mansoura 35516, Egypt; dr_amiraeladl@mans.edu.eg; 3 Microbiology and Immunology Unit, Department of Pathology, College of Medicine, Jouf University, Sakaka 72388, Saudi Arabia; itaher@ju.edu.sa; 4 Department of Pharmacology, College of Medicine, Horus University, Damietta 34511, Egypt; 5 Medical Histology and Cell Biology Department, Faculty of Medicine, Mansoura University, Mansoura 35516, Egypt; dodosands@yahoo.com (D.A.S.); monafarouk_2000@yahoo.com (M.F.M.S.); 6 Medical Microbiology and Immunology Department, Faculty of Medicine, Mansoura University, Mansoura 35516, Egypt

**Keywords:** *Escherichia coli*, ethanol, gut microbiota, *Klebsiella pneumoniae*, metformin, non-alcoholic fatty liver disease

## Abstract

Ethanol-producing dysbiotic gut microbiota could accelerate the progress of non-alcoholic fatty liver disease (NAFLD). Metformin demonstrated some benefits in NAFLD. In the present study, we tested the ability of metformin to modify ethanol-producing gut bacterial strains and, consequently, retard the progress of NAFLD. This 12-week study included forty mice divided into four groups (*n* = 10); normal diet, Western diet, Western diet with intraperitoneal metformin, and Western diet with oral metformin. Oral metformin has a slight advantage over intraperitoneal metformin in ameliorating the Western diet–induced changes in liver function tests and serum levels of different cytokines (IL-1β, IL-6, IL-17, and TNF-α). Changes in liver histology, fibrosis, lipid content, Ki67, and TNF-α were all corrected as well. Faecal ethanol contents were increased by the Western diet but did not improve after treatment with metformin although the numbers of ethanol-producing *Klebsiella pneumoniae* (*K. pneumoniae*) and *Escherichia coli* (*E. coli*) were decreased by oral metformin. Metformin did not affect bacterial ethanol production. It does not seem that modification of ethanol-producing *K. pneumoniae* and *E. coli* bacterial strains by metformin could have a significant impact on the therapeutic potentials of metformin in this experimental model of NAFLD.

## 1. Introduction

Non-alcoholic fatty liver disease (NAFLD) is a progressively increasing burden worldwide. It was estimated that 25–40% of elevated liver enzyme cases could be attributed to NAFLD [1]. Non-alcoholic steatohepatitis (NASH) term is used when simple fatty liver is associated with inflammation and fibrosis [2]. There is no current effective treatment for NASH and it is currently a major indication for liver transplantation [3].

For NAFLD/NASH to develop, it goes through a complex process. As reviewed elsewhere [4], several factors can contribute to the development and progress of NASH. Genetic susceptibility, mitochondrial dysfunction, oxidative stress, endoplasmic reticulum stress, immunological dysfunction abnormalities in lipid metabolism, and production of harmful cytokines have been all incriminated. In the past few years, dysbiosis of the gut microbiota gained much attention [5,6]. Dysbiosis of the gut microbiota can dysregulate appetite signalling and short-chain fatty acid production. It can also alter bile acid, choline, and amino acid metabolism, dysregulate the production of intestinal cytokines, and impair intestinal permeability [5,6]. In addition, it was suggested that these bacteria, especially certain strains of *Escherichia coli* (*E. coli*), have the ability to metabolise different carbohydrates and produce ethanol [7]. In addition to being absorbed and directly harmful to the liver, ethanol can impair the permeability of the intestine [8]. In a study conducted by Yuan et al., ethanol-producing strains of *Klebsiella pneumoniae* (*K. pneumoniae*) were identified in up to 60% of Chinese patients with NAFLD. Mice inoculation with these strains resulted in the development of NAFLD and their selective elimination prevented the development of the disease [9].

The antidiabetic drug, metformin, has shown some efficacy in managing NASH in several clinical and experimental studies [10,11,12,13]. Although such benefits were limited to biochemical parameters without reversing the disease [14], metformin is still one of the limited options in managing NAFLD. One of the suggested mechanisms by which metformin can produce its effects on NASH was its ability to modify the gut microbiota in favour of reducing NASH [11,13]. However, previous studies focused on the ability of metformin to prevent the loss of tight junction proteins, translocation of bacterial endotoxins, and restore the balance of gut microbiota. None of these studies considered the effect of metformin on ethanol-producing gut microbiota although these bacteria have been linked to the development of NAFLD [7,9]. Hence, the present study was conducted to investigate the ability of metformin to specifically modify certain ethanol-producing gut bacteria and to evaluate if such an effect could eventually affect the progress of NAFLD. The study focused on two ethanol-producing gut bacterial strains; *K. pneumoniae* and *E. coli*.

## 2. Results

### 2.1. Animal Weight

**Figure 1:**
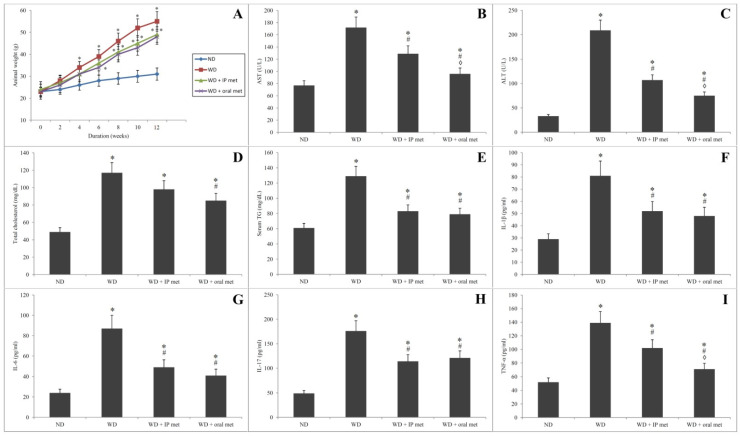
Changes in the weight of mice over 12 weeks after administration of Western diet with or without oral/IP metformin (**A**). Western diet resulted in a significant elevation of the serum levels of AST (**B**), ALT (**C**), total cholesterol (**D**), and serum triglycerides (**E**). Both oral and IP metformin were able to ameliorate these changes. The oral route had better outcomes for AST and ALT levels. For inflammatory markers, the Western diet resulted in a significant elevation of the serum levels of IL-1β (**F**), IL-6 (**G**), IL-17 (**H**), and TNF-α (**I**). Except for TNF-α, oral and IP metformin were equally effective in reducing the elevated serum levels of these cytokines in comparison to the Western diet only (*n* = 10 for each group; * significant (*p* < 0.05) from the control group; # significant from the Western diet only group, ◊ significant from the Western diet + IP metformin group; IP: intraperitoneal; Met: metformin; ND: normal diet; TG: triglycerides; WD: Western diet).

Using a Western diet to induce NAFLD resulted in a significant increase in the animals’ weight by week 4 of the experiment. Although the changes of weight in comparison to control were delayed to the 6th and 8th weeks for intraperitoneal (IP) and oral metformin, respectively, all the three groups, which were fed on the Western diet, did not show any significant difference in comparison to one another (Figure 1A).

### 2.2. Biochemical Parameters

The Western diet resulted in a significant increase in the serum levels of ALT and AST. Metformin significantly ameliorated these changes with some advantages of an oral route over the IP route (Figure 1B,C). For total cholesterol, only oral metformin was able to significantly reduce the elevation of serum total cholesterol in comparison to the Western diet group (Figure 1D). Both oral and IP metformin were able to reduce the elevated serum levels of triglycerides in comparison to the Western diet (Figure 1E).

### 2.3. Assay of Serum Cytokines’ Levels

Serum levels of all the tested cytokines (IL-1β, IL-6, IL-17, and TNF-α) were significantly increased by the Western diet. Both oral and IP metformin were capable of reducing the elevated serum levels of all cytokines in comparison to the group which received the Western diet only (Figure 1F–I). The only exception was the serum levels of TNF-α where oral metformin has some advantage over the IP metformin-treated group (Figure 1I).

### 2.4. Histological Results

**Figure 2:**
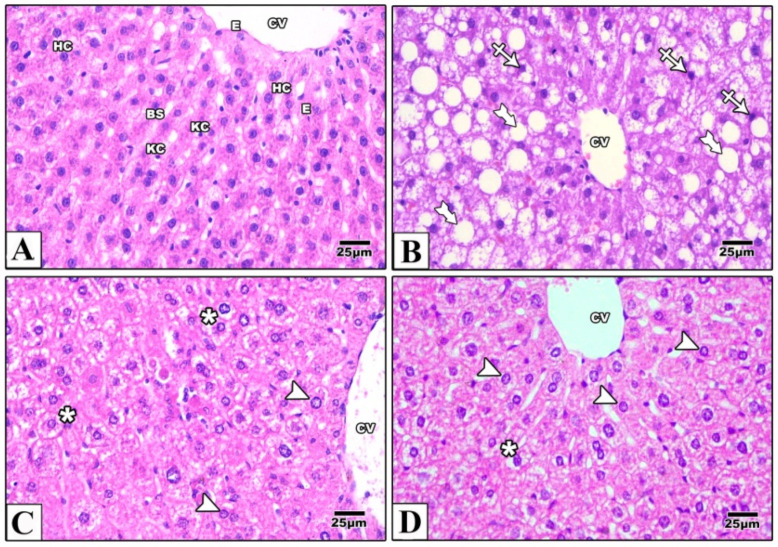
Photomicrographs of a paraffin section in the liver of the control group (**A**) showing that the hepatocytes (HC) were arranged in anastomosing plates radiating from the central veins (CV) lined by endothelial cells (E) and separated by blood sinusoids (BS) lined with Von Kupffer cells (KC). The polyhedral hepatocytes had acidophilic finely vacuolated cytoplasm and central rounded open-face nuclei. Western diet (**B**) resulted in degeneration of the hepatocytes (crossed arrow) and macrovesicular steatosis (tailed arrows). In IP (**C**) and oral (**D**) metformin, Western diet-induced changes were attenuated. Some hepatocytes showed vacuolations (asterisks) while others were normal (arrowheads). Magnification = ×400.

**Figure 3:**
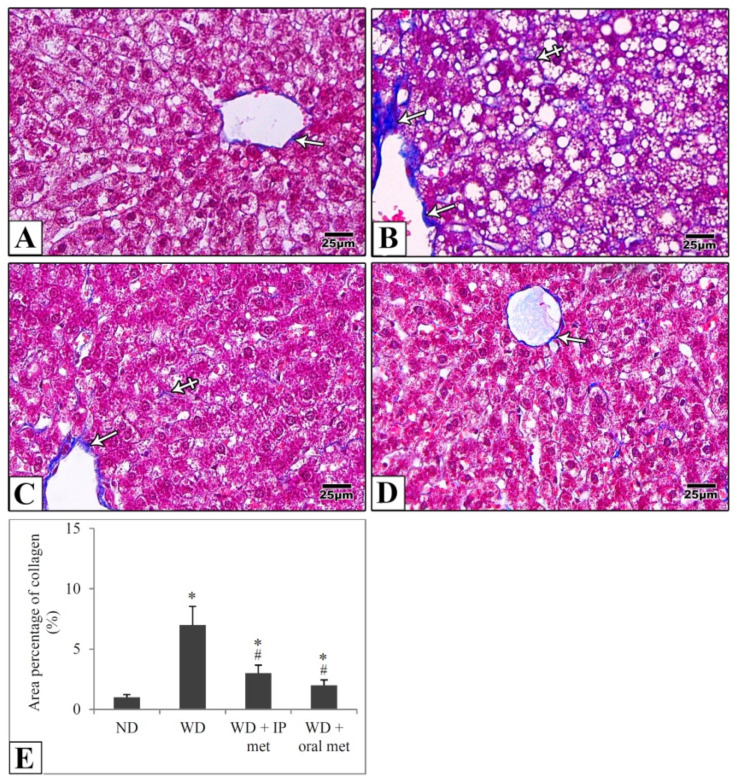
Representative histological images (**A**–**D**) and quantitative analysis (**E**) of collagen deposition. Paraffin sections from the mouse liver were stained with Masson’ Trichrome. Collagen deposition in the control group is mainly around the central vein (arrow) (**A**). Using the Western diet (**B**) resulted in extensive collagen deposition around the CV (arrow) and in between the hepatocytes (crossed arrows). Collagen deposition was much more reduced in IP (**C**) and oral (**D**) metformin groups. Area percentages of collagen deposition were presented in the chart (**E**). Magnification = ×400. * significant (*p* < 0.05) from control group; # significant from Western diet only group; IP: intraperitoneal; Met: metformin; ND: normal diet; WD: Western diet.

**Figure 4:**
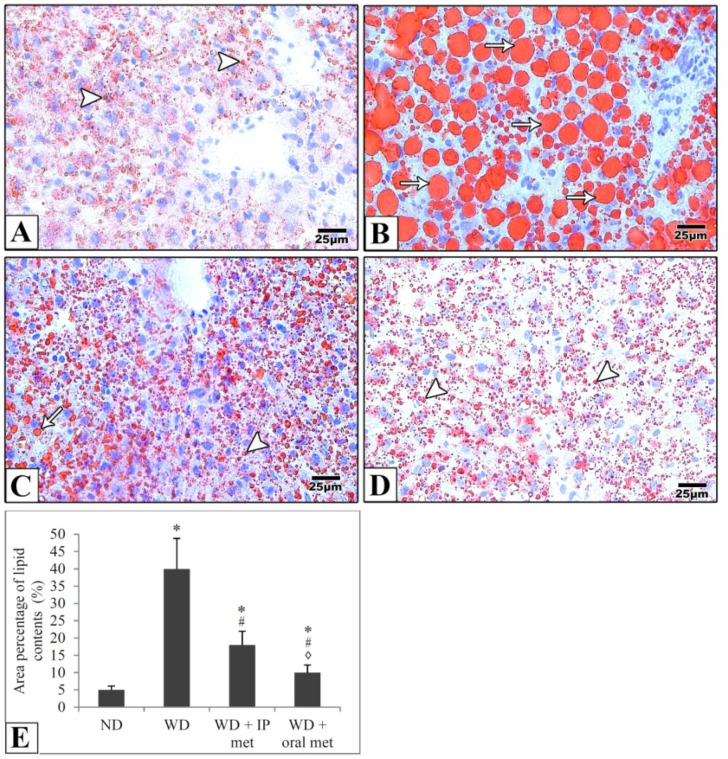
Representative histological images (**A**–**D**) and quantitative analysis (**E**) of hepatocytes lipid droplet contents. Photomicrographs of frozen sections from the liver stained with Oil Red O stain show that hepatocytes show few lipid droplets (arrowheads) in the control group (**A**). Large fat globules (arrows) were evident in the Western diet group (**B**). Lipid droplets were reduced in IP (**C**) and oral (**D**) metformin groups. Oral metformin outweighed IP metformin as indicated in the area percentages of lipid contents (**E**). Magnification = ×400. * significant (*p* < 0.05) from the control group; # significant from the Western diet only group, ◊ significant from the Western diet + IP metformin group; IP: intraperitoneal; Met: metformin; ND: normal diet; WD: Western diet.

In liver samples obtained from the control mice, hematoxylin and eosin-stained paraffin sections showed the characteristic arrangement of hepatocytes. Cells were arranged in anastomosing plates which radiate from the central veins, lined by endothelial cells, and separated by blood sinusoids which were lined by Von Kupffer cells. The polyhedral hepatocytes had acidophilic finely vacuolated cytoplasm and central rounded open-face nuclei. Some hepatocytes were binucleated. In the Western diet group, the hepatocytes showed degeneration and macrovesicular steatosis. Both metformin-treated groups showed that some hepatocytes exhibited vacuolations. Otherwise normal-looking hepatocytes were also observed (Figure 2). For Masson’s trichrome stain, the control group revealed thin rims of collagen deposited around the central veins and sinusoids. Using the Western diet alone resulted in an extensive deposition of collagen around the central veins and in between the hepatocytes. Treatment with IP or oral metformin resulted in less collagen deposition as compared to the Western diet alone (Figure 3). Steatosis and hepatocytes lipid contents were assessed by Oil Red O stain. In the control group, the hepatocytes showed few lipid droplets. In comparison, the group which was fed the Western diet showed large fat globules. Treatment with metformin resulted in a significant reduction in the number of lipid droplets with some advantages for the oral route (Figure 4).

### 2.5. Immunohistochemical Staining

**Figure 5:**
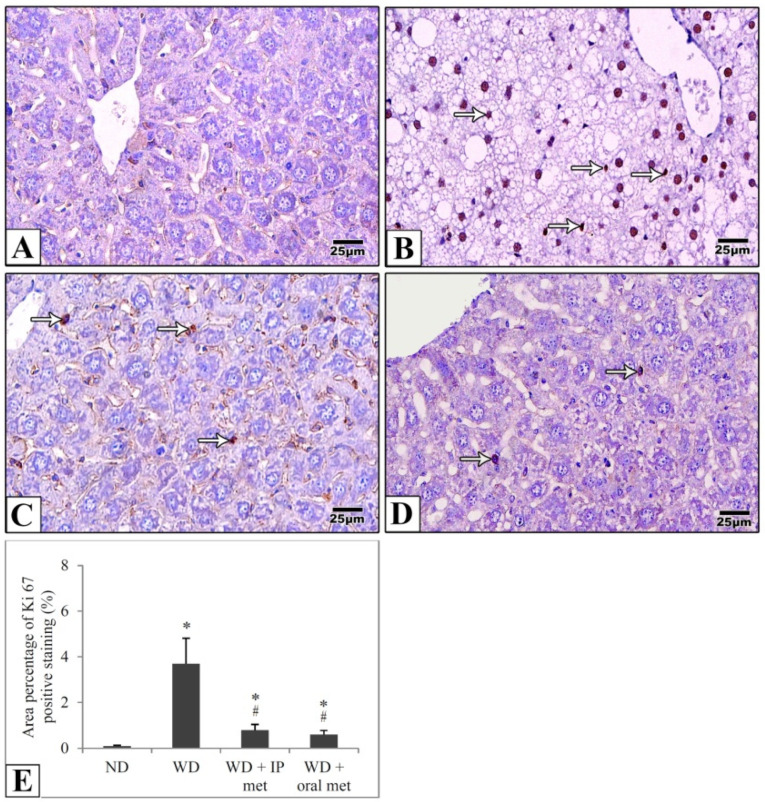
Representative histological images (**A**–**D**) and quantitative analysis (**E**) of Ki67 expression in immunostained liver sections in mice to detect cellular proliferation. Negative expression was evident in the control group (**A**) in comparison to the strong positive expression (arrows) in the Western diet group (**B**). Ki67 expression was significantly attenuated in IP (**C**) and oral (**D**) metformin groups. The area percentages of Ki67 expression for all four groups were also presented (**E**). Magnification = ×400. * significant (*p* < 0.05) from control group; # significant from the Western diet only group; IP: intraperitoneal; Met: metformin; ND: normal diet; WD: Western diet.

**Figure 6:**
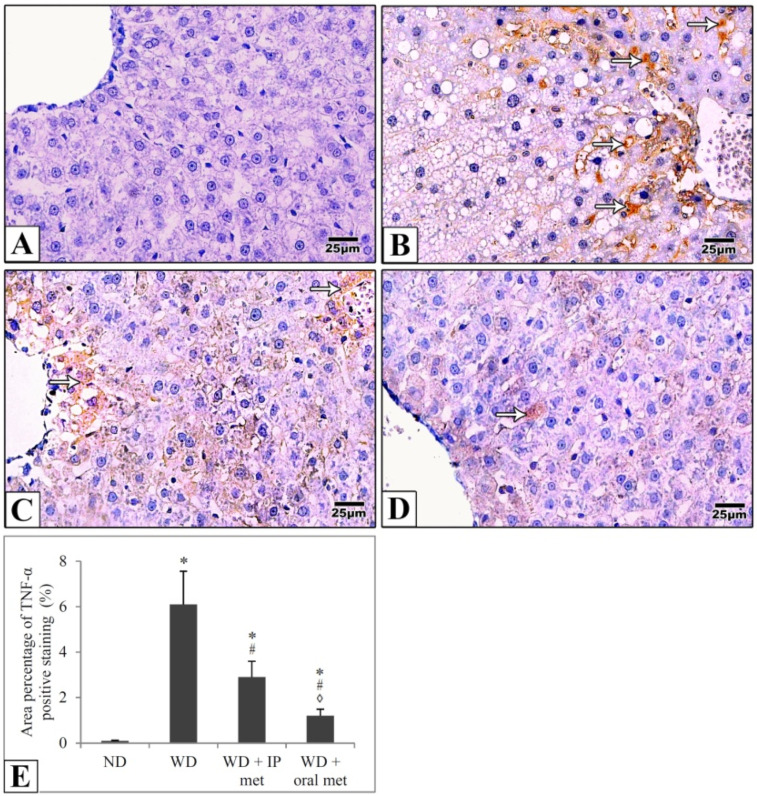
Representative histological images (**A**–**D**) and quantitative analysis (**E**) of TNF-α expression in immunostained liver sections in mice. TNF-α expression (arrows) was weak in the control group (**A**) in comparison to the Western diet group (**B**). TNF-α expression was significantly attenuated in IP (**C**) and oral (**D**) metformin groups. The area percentages of TNF-α expression for all four groups were also presented and the effect of oral metformin outweighed that of IP metformin (**E**). Magnification = ×400. * significant (*p* < 0.05) from the control group; # significant from the Western diet only group, ◊ significant from the Western diet + IP metformin group; IP: intraperitoneal; Met: metformin; ND: normal diet; WD: Western diet.

Sections of the liver of all studied groups were stained by immunohistochemistry using antibodies against Ki67 and TNF-α. Negative expression of Ki67 was evident in the control group in comparison to strong positive expression in the western diet group. Treatment with either IP or oral metformin resulted in a comparable significant reduction of Ki67 expression (Figure 5). For TNF-α expression, the Western diet group showed a relatively strong positive expression. Again, oral metformin outweighed the IP metformin in ameliorating the enhanced TNF-α expression (Figure 6).

### 2.6. Stool Analysis for Ethanol Contents

**Figure 7:**
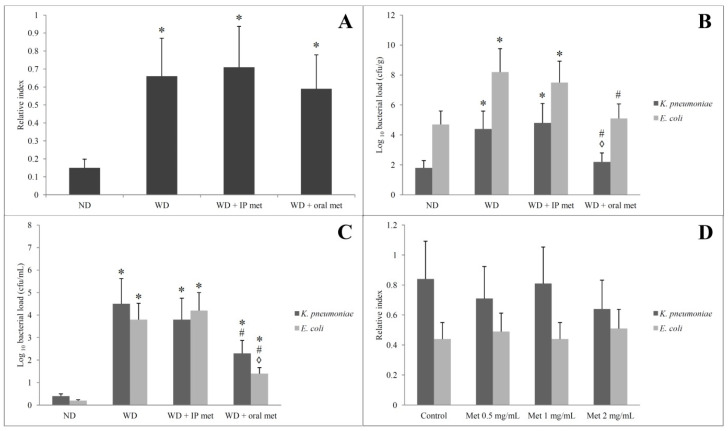
Effect of oral or IP metformin on the faecal ethanol contents of mice fed on Western diet over 12 weeks (**A**). Load of *K. pneumoniae* and *E. coli* (**B**) and their ethanol-producing isolates (**C**) in the stool of mice fed on a Western diet for 12 weeks with or without oral/IP metformin treatment. Bacterial loads (**B**,**C**) were compared between groups but for the same isolate (either *K. pneumoniae* or *E. coli* but not against each other). Upon in vitro cultivation, the ethanol production from the ethanol-producing isolates of *K. pneumoniae* and *E. coli* was unaffected by the addition of different concentrations of metformin (**D**). (* significant (*p* < 0.05) from the control group; # significant from the Western diet only group, ◊ significant from the Western diet + IP metformin group; IP: intraperitoneal; Met: metformin; ND: normal diet; WD: Western diet).

Faecal ethanol contents were relatively quantified in comparison to diethyl acetic acid. Faecal ethanol contents were elevated in all groups which were fed on a Western diet. Metformin, by either IP or oral routes, did not significantly affect the amount of ethanol in the stool (Figure 7A).

### 2.7. Selective Isolation of Ethanol-Resistant K. pneumoniae and E. coli

Culturing samples obtained from the animal faeces directly on the MacConkey agar plate revealed many lactose fermenting colonies. The colonies of *K. pneumoniae* were mucoid. The pure colonies of each bacterial isolate were identified by standard microbiological methods including Gram stain and biochemical reactions. The *K. pneumoniae* and *E. coli* isolates (Gram-negative bacilli, and lactose fermenters) were confirmed by the Vitek-2 compact system. Adding 5% and 10% ethanol to the bottles that contain MacConkey broth was carried out to identify alcohol-resistant non-fastidious Gram-negative bacteria. *K. pneumoniae* and *E. coli* isolates resistant to 5% ethanol were identified in all samples from the 4 tested groups. No isolate was resistant to 10% ethanol. Quantification of the number of colonies of *K. pneumoniae* and *E. coli* was conducted on 2 levels. First, samples cultivated directly from stool showed a significant increase in the number of colonies of *K. pneumoniae* and *E. coli* in the Western diet-treated group. Only the oral metformin group was able to reduce the number of colonies of both *K. pneumoniae* and *E. coli* (Figure 7B). The second level was to count ethanol-producing *K. pneumoniae* and *E. coli*. These isolates were obtained by growing the faecal samples on MacConkey broth with 5% ethanol and inoculating a fixed volume of the bottle content on MacConkey agar on day 3. Again, the Western diet resulted in a significant increase in the number of colonies of *K. pneumoniae* and *E. coli* and only the oral metformin group was able to reduce the number of colonies of both *K. pneumoniae* and *E. coli* (Figure 7C).

### 2.8. Alcohol Dehydrogenase (ADH) Gene Amplification by PCR and Measurement of Bacterial Ethanol Production in the Presence of Metformin

Adh gene was identified in ethanol-resistant *K. pneumoniae* and *E. coli* isolates in all samples which were obtained from the 4 tested groups confirming alcohol production. Biochemically, ethanol production from the isolated strains of *K. pneumoniae* and *E. coli* was confirmed by gas chromatography/mass spectrometry. Using three different concentrations of metformin did not significantly affect the ethanol production by the specified isolates (Figure 7D).

## 3. Discussion

NAFLD and NASH are worldwide health problems that have no current effective treatment [1]. One of the drugs that have been tested to treat NAFLD was metformin. In fact, the drug showed promising results in both experimental and clinical studies [10,11,12,13]. As reviewed elsewhere [15,16], the beneficial effects of metformin in NAFLD were attributed to several mechanisms. As dysbiosis of the gut microbiota was recently suggested as a key player in NAFLD development or progress [5,6], several studies suggested that the benefits of metformin in NAFLD could be partially attributed to the modification of such gut dysbiosis. Preservation of the duodenal tight junction proteins, reduction of bacterial endotoxin translocation, and restoring the disturbance of short-chain fatty acid balance were all suggested as potential mechanisms [11,13].

In the past few years, experimental and clinical evidence of the role of some ethanol-producing strains, namely *K. pneumoniae*, in the gut microbiota as a contributing factor to the development of NAFLD has gained much attention [7,9]. To test whether metformin can modify this particular point, we used two approaches. The first one was comparing oral versus IP metformin in mice models of NASH. The other was to directly evaluate the drug’s ability to modify alcohol-producing *K. pneumoniae* in stool and the reflection of such effect on total faecal ethanol contents. We were able to demonstrate that although metformin has a significant ability to prevent the development of NAFLD in mice models, none of these beneficial effects could be attributed to the reduction of ethanol production by the gut bacteria. Although the use of metformin reduced the number of ethanol-producing strains of *K. pneumoniae* and *E. coli* in the gut, the overall ethanol content in the gut was unchanged.

Comparing the oral and IP routes of metformin revealed that there is a slight advantage of using an oral route to administer metformin in certain parameters (AST, ALT, TNF-α, lipid contents in the hepatocytes). As reviewed by LaMoia & Shulman [17], the primary metabolic effects of metformin did not show much difference when oral or systemic administration of metformin was used. Many mechanisms which explain the benefits of metformin in NAFLD were attributed to its systemic effects [15,16]. A major mechanism that could explain the metabolic benefits of metformin is through the activation of AMP-activated protein kinase (AMPK) [18]. Improving insulin sensitivity, anti-inflammatory effects, reduction of TNF-α, inducible nitric oxide synthase (iNOS), interleukin1β, and antioxidant effects have been also suggested [13,19,20,21]. The apparent explanation of these results could suggest that the modification of gut microbiota by metformin is not a key player in the prevention of NAFLD. Although the model was somewhat different from NAFLD, it was reported that the metabolic benefits of metformin in mice model of metabolic syndrome were not significantly affected by the use of IP metformin or by reduction or elimination of gut microbiota using antibiotics or germfree mice respectively [22]. Modification of gut microbiota by metformin [11,13] might act as an add-on mechanism and explain the slight advantage of oral metformin. In fact, it was suggested that systemically administered metformin might indirectly impact the gut microbiota [22]. Another factor that should not be overlooked is related to the doses of metformin used in the oral route versus the IP route. We first tried equal doses of metformin for both routes (300 mg/kg/day) but almost all mice in the IP arm died before completing the 6th week. Selection of the doses in the present study was based on reviewing several experimental studies [22,23]. The differences in IP and oral route in these studies probably took into account the bioavailability of metformin. Still, if part of the action is related to the drug action on the gut microbiota, the oral route in this situation can have an additional advantage. Such a point would be almost impossible to control.

A more precise approach was to measure the effect of metformin on faecal ethanol contents and the ethanol-producing microbiota. First, we focused on the isolation and quantification of ethanol-producing strains of *K. pneumoniae*. The use of oral metformin, rather than IP, was able to reduce the number of this bacterial strain in the gut. However, we could not demonstrate a significant impact of oral metformin on the overall ethanol content in the gut. For this reason, we decided to re-evaluate the faecal samples for ethanol-producing strains of another bacterium; *E. coli*. Again, the use of oral metformin resulted in a decrease in the number of *E. coli* in the gut. Of note, IP metformin did not show a similar effect on the tested bacterial strains. Similar to oral metformin, IP metformin did not change the ethanol content in the gut.

A review of the previous literature in order to explain our results was even more contradicting. In vitro testing of metformin against Klebsiella did not show any direct suppressant effect [24]. Similarly, Wu et al. [25] reported that metformin did not directly promote the growth of *E. coli* in pure cultures. It seems that the effect of metformin on these bacteria would be more relevant to modifying the overall gut microbiota [24,25]. Feeding rats on high fat for 3 days only showed a significant increase in the abundance of *K. pneumoniae* and *E. coli* in the upper small intestine. Pre-treatment with metformin for one day did not have a significant impact on the abundance of these strains. However, high fat-fed rats transplanted with metformin-treated microbiota exhibited a significant reduction in *E. coli* while *K. pneumoniae* was not tested [26]. Zhang et al. [27] reported that using metformin for 8 weeks in highly fat-fed rats resulted in the enrichment of the genus Klebsiella. For *E. coli*, most of the experimental studies in mice or rats used metformin with a high-fat diet and reported a significant reduction of *E. coli* [28,29,30,31]. In humans, we could not find any data regarding the effect of metformin on gut microbiota in NAFLD or NSAH patients. The use of metformin in patients with type 2 diabetes resulted in contradicting results for Klebsiella [25,32,33]. For *E. coli*, the results were more consistent. Most of the studies reported that using metformin could result in a significant increase in the abundance of *E. coli* in the gut either in human patients with diabetes [25,32,34] or even healthy adults [35]. Several confounding factors could explain the variation between experimental and clinical studies and is difficult to control. Even in clinical studies, the results vary depending on the sample size, dose, study duration, race, and disease status [36].

Nevertheless, affection of the bacterial abundance number is not the only effect of metformin. The drug could also affect the metabolic activity of these bacteria. One example is trimethylamine N-oxide (TMAO). TMAO is a hazardous gut microbial metabolite that plays a role in NAFLD [37]. It was reported that the use of metformin in db/db mice resulted in a significant reduction in the plasma levels of TMAO. In vitro testing revealed that metformin significantly decreased the bacterial production rate of the precursor of TMAO. The tested bacteria were *K. pneumoniae* and *Proteus mirabilis* [38]. For this reason, we decided to test whether ethanol production in the bacterial isolates could be affected by metformin. Using different concentrations of the drug did not significantly impact ethanol production by either *K. pneumoniae* or *E. coli*.

An explanation for our bizarre results came from a very recent work of Mbaye et al. [39]. In their study, they found that the faecal ethanol contents were four times higher in NASH patients in comparison to control. Many strains of yeasts were isolated from the faeces of NASH patients but not from controls. In vitro testing revealed that the isolated yeast strains were able to metabolise fructose into ethanol in concentrations that were ten times higher than ethanol produced by bacteria. They suggested that ethanol production is not limited to one bacterial strain and other bacteria and yeasts could be linked to the development of NASH. Of note, fructose has been added to the drinking water in our model to enhance the development of NAFLD. We believe that using a different model of the disease or different diet composition could lead to different results. To our knowledge, there are no studies that investigated the effect of metformin on gut yeasts. Another clinical study reported that inhibition of alcohol dehydrogenase in patients with NAFLD induced a 15-fold increase in peripheral blood ethanol concentrations. Such effect was abolished by the use of oral antibiotics for one week. However, the authors found that only Lactobacillaceae could be correlated with postprandial peripheral ethanol concentrations [40]. While the study of Yuan et al. [9] accused ethanol-producing *K. pneumoniae* only in the development of NAFLD, we believe that there no single gut organism nor even single common gut microbiota diversity could be accused in ethanol production in NAFLD. Thus, we should not expect a uniform effect of metformin in all NAFLD patients or to get consistent results in experimental and clinical studies.

Although our overall results indicated that the beneficial effect of metformin in NAFLD is not largely affected by ethanol-producing bacteria in the gut, our study has some limitations. First and for several technical reasons, we could not use the 16s rRNA sequencing to provide full mapping of gut bacterial and yeast diversity. That is why we did not provide an explanation for our finding that oral metformin reduced some of the ethanol-producing bacteria in the gut but did not affect the overall faecal ethanol contents. Moreover, counting of bacteria relied on counting the colonies which is considered a rough estimation in comparison to 16s rRNA sequencing which can estimate the real-time relative abundance of bacterial strainsin the gut. The other limitation is that we did not evaluate a possible indirect interaction between ethanol and metformin away from gut microbiota. While ethanol may participate in NAFLD by impairing the intestinal epithelial barrier permeability [41], metformin can restore the altered intestinal permeability and prevent endotoxin translocation [11].

## 4. Materials and Methods

### 4.1. Animals

All procedures and animal handling were in accordance with the US National Research Council’s “Guide for the Care and Use of Laboratory Animals”. The experimental protocol was approved by the Local Committee of Bioethics, Jouf University (approval number 30-06-42). Forty 6–8 week-old pathogen-free male C57BL/6 mice were involved in the study. All mice went through an adaptation period. An air-conditioned room with a light/dark cycle was used for housing. Mice had free access to water and were fed a standard pellet diet for a week. Mice were then divided into four groups:

1. Group I (*n* = 10), Control group; animals were kept on a standard pellet diet.
2. Group II (*n* = 10), animals were fed a Western diet.
3. Group III (*n* = 10), animals were fed a Western diet with IP metformin (Sigma-Aldrich) (100 mg/kg/day).
4. Group IV (*n* = 16), animals were fed a Western diet with oral metformin (300 mg/kg/day in drinking water (1.5 mg/mL), assuming that the average water consumption is about 4 mL of water per day).

In groups II–IV, and in addition to the Western diet, fructose was added to the drinking water (42 g/L) to enhance the development of the NAFLD model [42]. The study was continued for 12 weeks. Animals were weighed on a weekly basis. By the end of the 11th week, fresh faecal samples were collected over 7 days. Mice were then euthanised by halothane overdose. Using heart puncture, blood samples were collected into dry tubes, centrifuged at 2500× *g* for 15 min at 4 °C, and stored at −80 °C. The liver was also collected for further analysis.

### 4.2. Biochemical Parameters

Specific ELISA kits were used to measure serum levels of ALT and AST (Cusabio, Houston, TX, USA; Cat. CSB-E16539m and CSB-E12649m, respectively). A specific enzymatic colorimetric method was used to measure total cholesterol and serum triglycerides (Biodiagnostic, Giza, Egypt; Cat. No CH 12 20 and TR 20 30, respectively). All measurements were conducted according to the manufacturer’s instructions.

### 4.3. Assay of Serum Cytokines’ Levels

The serum levels of interleukin IL-1β, IL-6, IL-17, and TNF-α were measured using specific ELISA kits (Cusabio, Houston, TX, USA; Cat. CSB-E08054m; CSB-E04639m; CSB-E04608m and CSB-E04741m, respectively). All measurements were conducted according to the instructions of the manufacturer.

### 4.4. Histological Study

For the histological study, liver specimens were fixed in a neutral buffered formalin solution (10%) for 48 h. Ascending grades of alcohol were then used for dehydration. Liver specimens were cleared in xylene and then embedded in paraffin. Five-μm sections were cut by the use of a rotary microtome, mounted on glass slides, and then rehydrated through descending grades of alcohol. Staining with hematoxylin and eosin was used to identify the liver histology while Masson’s trichrome was used to assess collagen deposition. Frozen sections were processed and stained by Oil Red O stain to assess the steatosis [43,44].

### 4.5. Immunohistochemical Study

Five μm sections from the paraffin blocks were cut and mounted in charged slides. Sections were deparaffinised and rehydrated through a graded series of alcohol solutions. Sections were then incubated in antigen retrieval which was achieved by boiling the sections with sodium citrate buffer (0.01 mol/L, pH 6) for 10 min, then treated with 3% hydrogen peroxide to block endogenous peroxidase activity. Immunohistochemical staining was performed by the avidin–biotin immunoperoxidase method. Primary antibodies Ki67 rabbit polyclonal antibody (1:50 dilution; Novus Biologicals, Centennial, CO, USA; catalogue no. NB500-170SS) and anti-TNF-α (1:100 dilution; Abcam, Cambridge, MA, USA; catalogue no. Ab6671) were applied on the slides and incubated overnight in a refrigerator at 4 °C. Secondary biotinylated antibody was then applied, followed by incubation with streptavidin peroxidase (Dako Corp., Carpinteria, CA, USA.). Sections were washed with phosphate buffer saline (PBS) three times after each step. Sections were then stained with diaminobenzidine chromogen solution (DAB), and counterstained with Mayer’s hematoxylin [45,46], then dehydrated and mounted. Negative control sections were obtained by omitting the primary antibodies.

### 4.6. Morphometric Studies

In each group, ten randomly selected non-overlapping microscopic fields/mice were photographed. The used camera was Olympus digital camera (E24-10 megapixel) built into an Olympus microscope with a 0.5× photo adaptor using the 40× objective. Data were then analyzed by the use of Fiji ImajeJ software. Area percentages of collagen, Ki67, and TNF-α were analysed in the ten chosen fields. For Masson’s trichrome-stained sections, the mean area percentage (%) of collagen fibre content was calculated after adjusting the colour threshold and excluding the background. The following formula was used to calculate the selected blue-stained area: Area percentage (%) = (blue-stained area/total field area) × 100. The same approach was adopted for Oil Red O staining. Similarly, the mean areas % of Ki67 and TNF-α immunostaining were calculated.

### 4.7. Stool Analysis for Ethanol Contents

Seven days before animals were sacrificed; fresh faecal samples were collected daily and were immediately frozen at −80 °C. Measurement of ethanol content in mice faeces was conducted according to a previously described method for the evaluation of volatile organic compounds in faeces with minor modifications [47,48]. In brief, an Agilent 6890 gas chromatography equipped with an Agilent mass spectrometric detector (Agilent Technologies, Santa Clara, CA, USA). Faecal samples (125 mg) were suspended in 5 mL of water in Headspace glass vials. The internal standard was diethyl acetic acid (1.5 mg/L). A magnetic stirrer, sulphuric acid, and a pinch of sodium sulphate were added to the sample to acidify and salt out the solution. Ethanol was then relatively quantified compared with diethyl acetic acid.

### 4.8. Selective Isolation of Ethanol-Resistant K. pneumoniae and E. coli

From the previously frozen faecal samples, 250 mg of faeces was suspended in 1 mL of PBS 1X. Glass beads were used to vortex the suspension which was then incubated at 4 °C for 2 h and centrifuged at 80× *g* for 1 min to remove debris as a pellet [49]. A 50 μL of the supernatant was cultured on MacConkey (Oxoid, Basingstoke, UK) agar plate and incubated aerobically at 37 °C for 48 h. Viable colonies were then counted and results were expressed as log10 of colony forming units (CFU) per gram of stool (cfu/g). In a parallel approach, we used blood culture bottles (Oxoid, Basingstoke, UK) previously emptied of their contents. 20 mL of MacConkey broth (Oxoid, Basingstoke, UK) enriched with 4 mL of defibrinated sheep blood and 4 mL of sterile rumen juice were added to the bottles. 200 μL of the initial suspension (250 mg of stools in 1 mL) of each sample was inoculated into the bottles and incubated aerobically for 10 days. On Day 1, Day 3, Day 7, and Day 10, 500 μL of the bottle content was sampled followed by 10-fold serial dilutions and inoculation on MacConkey agar. The same method was used but with the addition of 5% and 10% ethanol to the bottle in order to identify alcohol-resistant non-fastidious Gram-negative bacteria [39]. Counting in this approach was expressed as log10 of cfu/mL. All media included in this study were prepared according to the manufacturer’s instructions. The pure colonies of each bacterial isolate were identified by standard microbiological methods including Gram stain and biochemical reactions then counted. The *E. coli* and *K. pneumoniae* isolates (Gram-negative bacilli, and lactose fermenters) were confirmed by the Vitek-2 compact system using the Gram-negative identification (GN-ID) cards (BioMérieux, Marcy l’Etoile, France). For each isolate, triplicate QC testing was performed by using American Type Culture Collection (ATCC) strains (*E. coli*; ATCC10536, *K. pneumoniae*; ATCC10031) as positive controls.

### 4.9. Adh Gene of Ethanol-Resistant K. pneumoniae and E. coli; Amplification by PCR

The Vitek-2-confirmed ethanol-resistant *K. pneumoniae* and *E. coli* isolates were tested for the *Adh* gene by PCR. Briefly, bacterial chromosomal DNA was extracted according to the method described by Wilson [50] to get the DNA templates. *Adh* gene amplification by PCR was conducted using a pair of primers (Sigma) selected according to [9,51] & the sequence of the primer used was:

F: 5′-ATGAAGTATGTGAATCTGGG-3′

R: 5′-TTAATAGTTCTGGATCGCTG-3′

The PCR reaction was conducted in a final volume of 25 μL which contained:

1. A volume of 12.5 μL of Taq PCR Master Mix after being briefly vortexed to avoid localised differences in salt concentration.
2. The primer solutions were thawed on ice & mixed well before use. One μL of each primer was added to the PCR tube.
3. A volume of 5 μL of template-extracted DNA was added to each tube.
4. A volume of 5.5 μL of nuclease-free double distilled water was added.

The thermal cycler program was adjusted & preceded as shown in Table 1 [52]:

Agarose gel (1.5%) electrophoresis of the amplified *Adh* gene was conducted using the DNA molecular marker (100 bp DNA Ladder; Lonza Inc., Rockland, MA, USA) to detect the expected (1038 bp) bands visualised by staining with ethidium bromide (EB) [53].

### 4.10. Measurement of Ethanol Production by Ethanol-Resistant K. pneumoniae and E. coli

The Vitek-2-confirmed ethanol-resistant *K. pneumoniae* and *E. coli* isolates were then tested for ethanol production. One mL of a bacterial suspension of 1.5 × 104 cfu/mL was inoculated into 20 mL of liquid MacConkey broth (Oxoid, Basingstoke, UK). The ability of each isolate to produce ethanol was then tested in the presence of 3 different concentrations of metformin (0.5, 1, and 2 mg/mL) in 6 technical replicates. After 24 h of incubation at 37 °C, two mL of each culture was placed in Headspace glass vials to measure the ethanol concentration by gas chromatography equipped with a mass spectrometric detector as described in the assay of faecal ethanol contents.

### 4.11. Data Analysis

Using SPSS software (ver. 22, SPSS Inc., Chicago, IL, USA), data were analyzed. The normal distribution of data was tested using Kolmogorov–Smirnov test. One-way ANOVA followed by a Bonferroni post hoc test for multiple comparisons was used and values are expressed as mean ± SD. A *p*-value < 0.05 is considered statistically significant.

## 5. Conclusions

The result of the current study indicated that the beneficial effects of metformin in a mouse model of NAFLD could not be attributed to the modification of the number or activity of ethanol-producing *K. pneumoniae* and *E. coli* in the gut. Still, full mapping of the gut microbiota, probably by using 16s rRNA sequencing technique, might provide a clear insight into the effect of modification of ethanol-producing bacteria on the progress of NAFLD. Considering the ethanol-producing gut yeast is also crucial. Finally, addressing these limitations in a well-designed clinical trial will be more relevant to the pathogenesis of NAFLD and gut microbiota diversity in humans.

## Figures and Tables

**Table 1:** The thermal cycler program for amplification and detection of *Adh* gene of ethanol-resistant *K. pneumoniae* and *E. coli* by PCR.

Step	Initial Denaturation	Three-Step Cycling × 30 Times:			Final Extension
		Denaturation	Annealing	Extension	
Duration	2 min	10 s	30 s	3 min	5 min
Temperature	94 °C	98 °C	60 °C	68 °C	68 °C

## Data Availability

The data supporting the findings of the article is available upon request from the author.
